# Boosting the impact of seasonal malaria chemoprevention (SMC) through simultaneous screening and treatment of household members of children receiving SMC in Burkina Faso: a protocol for a randomized open label trial

**DOI:** 10.1186/s13690-022-00800-x

**Published:** 2022-01-27

**Authors:** Paul Sondo, Marc Christian Tahita, Hamidou Ilboudo, Toussaint Rouamba, Karim Derra, Gauthier Tougri, Florence Ouédraogo, Béatrice Marie Adélaïde Konseibo, Eli Roamba, Sabina Dahlström Otienoburu, Bérenger Kaboré, Kalynn Kennon, Kadija Ouédraogo, Wend-Timbe-Noma Arlette Raïssa Zongo, Fadima Yaya Bocoum, Kasia Stepniewska, Mehul Dhorda, Philippe J. Guérin, Halidou Tinto

**Affiliations:** 1 Intitut de Recherche en Siences de la Santé (IRSS), Clinical Research Unit of Nanoro (CRUN), Nanoro, Burkina Faso; 2 Ministry of health of Burkina Faso, National Malaria Control Program, Ouagadougou, Burkina Faso; 3 Infectious Diseases Data Observatory (IDDO), Oxford, UK; 4 WorldWide Antimalarial Resistance Network (WWARN), Oxford, UK; 5 College of STEM, Johnson C. Smith University, Charlotte, North Carolina, USA; 6 Centre for Tropical Medicine and Global Health, Nuffield Department of Medicine, University of Oxford, Oxford, UK

**Keywords:** Malaria, Chemoprevention, *Plasmodium falciparum*, Africa, Burkina Faso, Amodiaquine, Sulfadoxine-pyrimethamine, Dihydro artemisinin Piperaquine

## Abstract

**Background:**

*Plasmodium falciparum* malaria remains a major public health concern in sub-Sahara Africa. Seasonal malaria chemoprevention (SMC) with amodiaquine + sulfadoxine-pyrimethamine is one of the most important preventive interventions. Despite its implementation, the burden of malaria is still very high in children under five years old in Burkina Faso, suggesting that the expected impact of this promising strategy might not be attained. Development of innovative strategies to improve the efficacy of these existing malaria control measures is essential. In such context, we postulate that screening and treatment of malaria in household members of children receiving SMC could greatly improve the impact of SMC intervention and reduce malaria transmission in endemic settings.

**Methods:**

This randomized superiority trial will be carried out in the Nanoro health district, Burkina Faso. The unit of randomisation will be the household and all eligible children from a household will be allocated to the same study group. Households with 3–59 months old children will be assigned to either (i) control group (SMC alone) or (ii) intervention (SMC+ screening of household members with standard Histidin Rich Protein Rapid Diagnostic Test (HRP2-RDT) and treatment if positive). The sample size will be 526 isolated households per arm, i.e., around 1052 children under SMC coverage and an expected 1315 household members. Included children will be followed-up for 24 months to fully cover two consecutive malaria transmission seasons and two SMC cycles. Children will be actively followed-up during the malaria transmission seasons while in the dry seasons the follow-up will be passive.

**Conclusion:**

The study will respond to a major public health concern by providing evidence of the efficacy of an innovative strategy to boost the impact of SMC intervention.

## Background

Malaria remains a major public health concern in sub-Sahara Africa. According to the World Health Organization (WHO) nearly half of the world’s population is exposed to malaria infection causing 409,000 deaths worldwide in 2019 [1]. In Burkina Faso like in most sub-Sahara Africa countries, malaria is endemic with peaks during rainy seasons. To reduce the burden of the disease in the country, Burkina Faso has subscribed to the Roll Back Malaria initiative and adopted several malaria control measures including the use of artemisinin-based combination therapies (ACTs) as first line treatment since 2005, intermittent preventive treatment in pregnancy (IPTp), wide-scale distribution of long-lasting insecticide treated nets, and seasonal malaria chemoprevention (SMC) for children younger than five years [2]. However, despite the implementation of these multiple interventions, Burkina Faso is ranked among the top 10 countries carrying the highest malaria burden. For instance, in 2019, over 10 million clinical episodes and 1060 deaths were reported in the country [1].

In this context, new strategies to complement or to improve the impact of ongoing interventions are urgently needed in order to reduce malaria transmission. The study we propose aims at improving the effect of Seasonal Malaria Chemoprevention (SMC) intervention to achieve best impact in malaria control and elimination. SMC is one of the most important and reliable malaria preventive measures recommended by WHO and is known to reduce malaria morbidity by 30–83% [3–5]. It involves the administration of a combination of antimalarials, amodiaquine with sulfadoxine-pyrimethamine (AQSP) to children aged 3–59 months on a monthly basis during the high transmission season [2]. In Burkina Faso, SMC is implemented nationally from July to October by the National Malaria Control Program (NMCP) with the support of its technical and financial partners such as Global Fund, WHO, Malaria Consortium, President’s Malaria Initiative (PMI), and United Nations International Children’s Emergency Fund (UNICEF). SMC was firstly implemented in 2014 in 6 of the 70 health districts of the country. In 2018, national wide coverage was almost reached (except in Ouagadougou i.e. SMC was implemented in 60 health districts over 70 in total) and the intervention was considered to be adequately delivered by community health workers [6]. Since the adoption of this strategy, data assessing the real-life impact of this intervention in the country are sparse. In 2018, Druetz et al. reported a protective effect of 62% against clinical malaria in Burkina Faso. This study highlights the potential adding value of this intervention for malaria control though this protective effect appears far below the highest threshold of 83% reduction of malaria incidence expected from SMC intervention [7, 8].

The burden of malaria in children under five years old suggests that the expected impact of this promising intervention is not achieved. Indeed, children under five years old represented the most affected population accounting for about 90% of malaria cases. Rapid Diagnostic Test (RDT) confirmed malaria cases in the country increased from 1,219,975 cases, to 1,487,954, and 1,509,931, in 2016, 2017, and 2018 respectively [1]. In view of the actual trend, it becomes obvious that despite the implementation of this strategy, the burden of the disease and associated mortality is still very high in children under five in Burkina Faso, suggesting that the expected impact from this intervention is not achieved. This raises questions about other unknown factors that can negatively affect the effectiveness of SMC intervention. At the same time, huge efforts aiming at preventing human-vector contact were deployed such as the large-scale distribution of insecticide treated bed nets (LLIN). The latter are distributed on the basis of two individuals per LLIN unit. However, more than two individuals per bedroom is commonly observed in rural settings in Burkina Faso and most bedrooms are not adequate for the fixation of several LLINs. In addition, the frequent mosquito bites before sleeping time and changing mosquito behaviour becoming more aggressive before and after sleeping time is of concern [9, 10]. The latter could explain a persisting malaria transmission within a relatively close proximity despite full LLIN coverage.

Asymptomatic carriers of *P. falciparum* infection do contribute to the malaria reservoir, especially in highly endemic regions such as Burkina Faso, but no strategies currently exist aimed at draining this reservoir. In such circumstances, family members (e.g. mother, father, siblings and other relatives) living in the same room or house with children receiving SMC could continually sustain the transmission cycle despite existing measures such as the use of LLIN. According to the Nanoro health demographic surveillance system, an average of 3 inhabitants per household was observed [11]. Published literature on sleeping behaviour reported that children mostly sleep with their mother or with mother + sibling [12]. Attempts to improve the impact of SMC intervention includes its extension to children under ten years old which showed promising results in Senegal [13]. However, parents and older siblings (over ten years of age) not covered by the SMC (implemented either in under 5 or extended to under 10) sharing the same habitat could be parasites reservoir continuously infecting the most vulnerable group under SMC coverage [9, 10]. This is particularly important in a context of low coverage and compliance to LLIN use. Indeed, according to the household survey results in 2018 from the World Malaria Report, 54.5% of the population in Burkina Faso had access to LLIN while only 44.1% had slept under LLIN the night before the survey [1]. This phenomenon of continuous infection of children from parents and elder siblings would obviously jeopardize the expected impact of the SMC intervention and the global effort to control the disease.

The presence of parasite reservoir within the community represents a challenge for malaria control and elimination with even sub-patent infection contributing to the maintenance of transmission [10]. However, the detection of the reservoir remains difficult given the low sensitivity of routine RDT for the detection of low and sub-patent parasitemia (parasitemia not detectable by mircroscopy or RDT but detectable by polymerase chain reaction).

Finally, drug pressure, especially in wide scale community-based intervention such as SMC, could lead to a selection of mutant strains with decreased sensitivity to the drugs used, which could compromise the long-term effectiveness of the intervention [14, 15]. Therefore, sustainability of any intervention relies on monitoring the selection of parasite mutations associated with drug resistance.

All these aspects support the idea of this study which stands on the assumption that the screening of household members of children receiving SMC could greatly greatly contribute to improve the impact of SMC intervention and reduce malaria transmission in endemic settings. The primary objective aims to improve the impact of SMC intervention in terms of reducing malaria morbidity and mortality in children under five years through a new strategy consisting in a screening and treatment of household members of SMC children. Secondary objectives include the assessment of the the tolerance and safety profile of the added treatment to SMC, the impact of the new strategy on the circulating parasite population as well as determinant such as adherence to treatment, acceptability, and cost-effectiveness of the strategy.

## Methods

### Study area

**Fig. 1:**
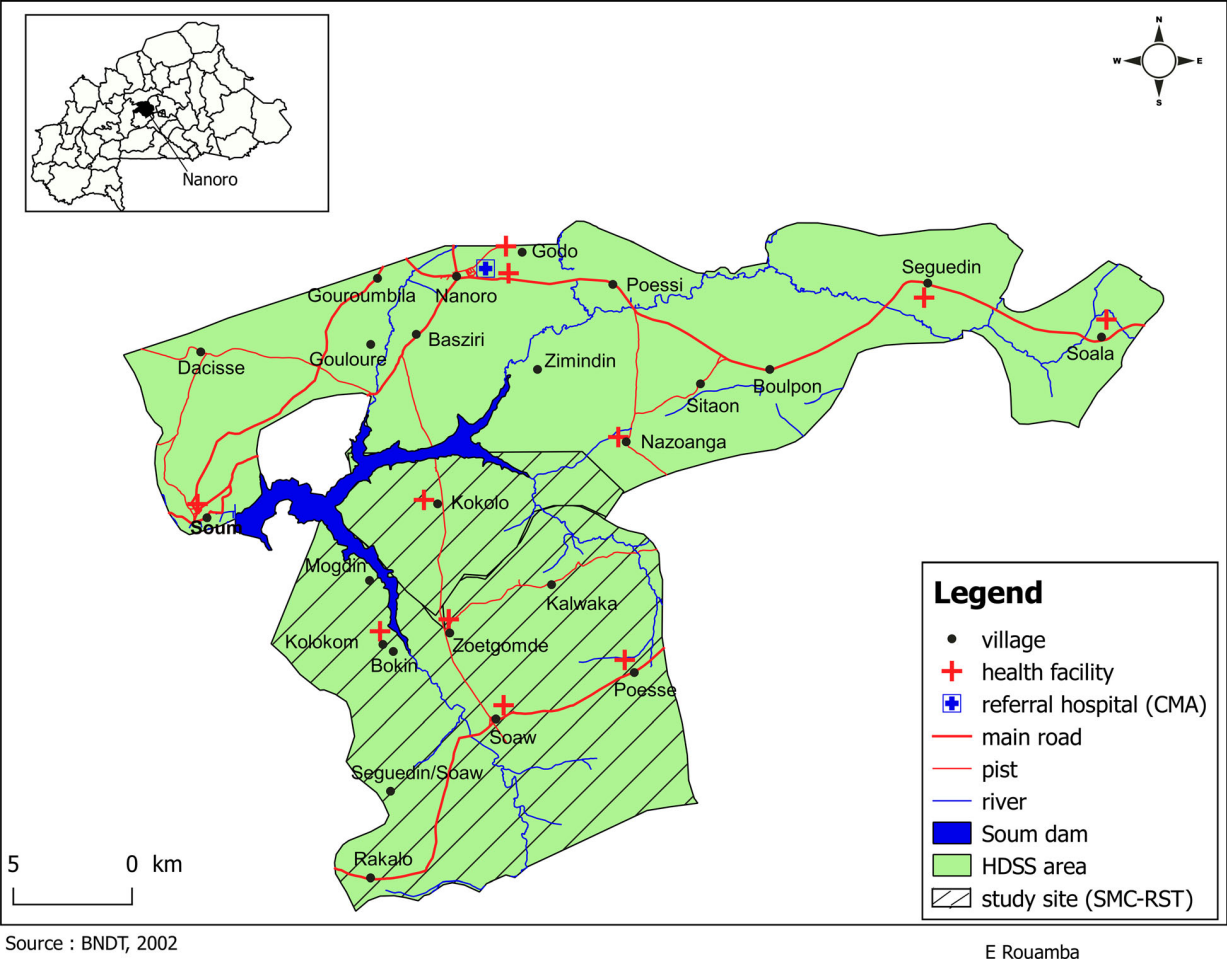
The study area within the Health district of Nanoro, Burkina Faso. Mapping of the Health District of Nanoro indicating the area where the study will be implemented

The study will be carried out in the Nanoro health district (NHD) catchment area which is situated in the central part of Burkina Faso. The NHD covers 5 departments: Nanoro, Soaw, Pella, Siglé and Kindi. The study will be implemented in the southern shore of the dam of Soum comprising the coverage area of 4 health facilities of the department of Soaw (Soaw, Zoetgomdé, Kologkom, and Poessé) and of one health facility (Kokolo) of the department of Nanoro. Figure 1 represents the area within the NHD that the study will cover (Fig. 1). The study area encompasses an area of 205,85 km^2^ with an approximate population of 19,915 inhabitants in 2018 according to the health and demographic surveillance system of the CRUN [11]. Like other Sahelian zones, the study area is characterized by high malaria risk during the rainy season [16], making the area an appropriate place for SMC implementation.

### Study design

**Fig. 2:**
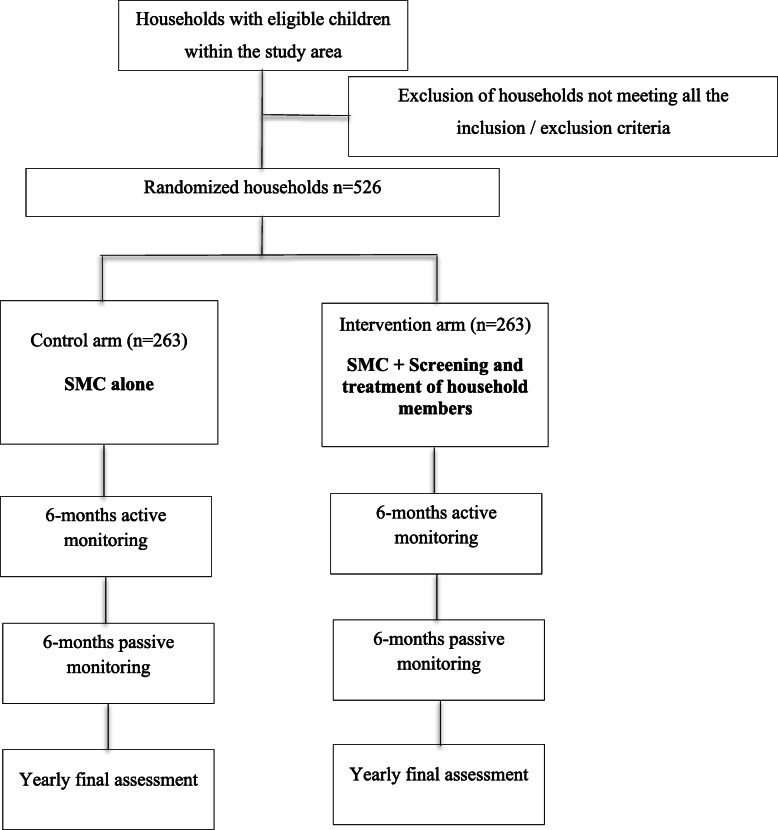
The trial profile indicating the flowchart of the study participant from enrolment in 2021 to close-out visit in 2023. Flowchart of the study participant from enrolment to close-out visit

This will be a randomized superiority trial in which children will be followed for two years. The unit of randomization will be the household and all eligible children from a household will be allocated to the same study arm to avoid confusion. The household will be defined as follows: one or several individuals living together within a shared habitat (house) and sharing basic needs, especially kitchen, and which recognizes the authority of a single person regardless of his/her gender. Households with children aged 3–59 months with at least one under 35 months of age living within the Nanoro Health and Demographic Surveillance System (HDSS) catchment area will be assigned to either (i) control group (SMC alone) or (ii) intervention (SMC+ household members screening with standard HRP2-RDT and treatment if positive). Figure 2 represents the trial profile highlighting the difference between the two study arms and key stages for the study implementation (Fig. 2).

### Study participants

The study population will be children under SMC coverage i.e. aged 3–59 months and members of their household (individuals of all ages) living within the Nanoro HDSS catchment area [10]. The intervention for this study will be the screening of households members (of children under SMC coverage) for malaria parasitaemia and treatment of positive cases with the aim to prevent continuous re-infections of children under SMC coverage from these reservoirs. Children will be followed-up for 24 months to cover two full consecutive malaria transmission seasons and two SMC cycles. Children who age out of the SMC program, i.e., whose age exceeds 59 months during the follow-up, will subsequently be considered as household members. Before the implementation of the year 1 first SMC round, a list of potential eligible households will be drawn from the HDSS database. Home visits will be performed to confirm the presence of the children and to assess willingness of the parents/guardians to participate in the study as well as willingness of all the household members to be screened and treated. Households with absent children or children whose parents/guardians and roommates are not willing to participate, will be replaced by other eligible households from the list. Enrolment of study participants will be made in parallel with the SMC first round after confirmation of the administration of the first dose of the SMC treatment.

### Inclusion/exclusion criteria

Inclusion criteria are: (i) Single household (not sharing the same concession with other households) with at least one child under 35 months of age under SMC coverage, (ii) household members residing within the HDSS catchment area, (iii) willingness of household members to be screened and treated, (iv) ability to complete the study follow-up period, and (v) written consent obtained from parents/guardian (vi) written consent/assent obtained from each household member.

Exclusion criteria are: (i) household with children under SMC coverage who did not receive the SMC (Amodiaquine-Sulfadoxine-Pyrimethamine) or sharing the same concession with other households, (ii) household with children under SMC coverage where at least one of his/her household members refuse to be screened and treated (these children will still receive the SMC treatment as part of their routine malaria prevention policy), (iii) severely ill individual at the time of enrolment including severe malaria (only individuals but household will be included), (iv) known allergy to AQSP for children and DHAPPQ for household members (the whole household will be excluded), (v) planned travel or inability to complete the study follow-up, (vi) participation in malaria vaccine trials or other therapeutic trials, and (vii) unwillingness to participate to the study.

### Sample size calculation

The aim of this study is to demonstrate that SMC for children between aged 3–59 months associated with the screening household members (through standard HRP2 RDT) and treatment if proven positive is superior to SMC alone in reducing the incidence of clinical malaria after 1 year. In this superiority trial the sample size is calculated to attain sufficient power to determine independently the relevant difference in malaria incidence rate between the control and the intervention arms, using classic RDT. The sample size estimation is performed under the assumption that the average number of malaria episodes per child aged between 3 and 59 months per year since the implementation of SMC in Nanoro health district varied between 1.38 and 1.76 [17–19]. Sample size of 236 in each arm will give at least 80% power to detect a 20% decrease in incidence of malaria after 1 year in comparison to a baseline incidence rate in the control arm (SMC) between 1.5–2.0 malaria cases per year, assuming a one-sided test with significance level of 0.025 and large samples z-test of the Poisson event rate difference (PASS software). To account for 10% study drop-out rate for any reason, the sample size will increase to 263 in each arm. Therefore, the final estimated sample size for this study is 526 households, with at least one child aged between 3 and 35 months to allow a follow up of the children during the study period and while benefiting of SMC. One child from each household (primary participants) will be included in the primary analysis, assuming that each household will have an average of 2 children under SMC coverage, about 1052 children under SMC coverage are expected [11]. The expected number of household members is estimated at about 1315 (263 × 5) under the assumption that each household from the intervention arm will have an average of 5 members. Data will be analysed with R software using the appropriate tests depending on the variables. Adherence to treatment and acceptability of the strategy (focus group discussion) will be assessed qualitatively.

### Assignment of interventions: allocation

Allocation of an household to a specific study arm will follow a predefined computer-generated randomization list. The list will be generated at the central level by the pharmacist of the CRUN. The randomization list will comprise a sequence of random numbers with corresponding study arm which will be sealed in an envelope. The envelope will only be opened by field workers after enrolment of the participating household. No blinding will be applied, making the study will be an open label trial.

### Data collection

The proposed study includes two major interventions: the already existing and funded SMC intervention by the National Malaria Control Program and the newly proposed intervention (Household members screening and treatment) expected to boost the impact of the SMC intervention. For SMC intervention, complete dose (3 days regimen) of Amodiaquine + sulfadoxine-pyrimethamine will be administered monthly over a period of four months. Each monthly administration will be performed according to children age as follow: [3–11] months, SP: 262,5 mg/AQ: 76,5 mg on day 0, SP: 0 mg/AQ: 76,5 mg on day 1, SP: 0 mg/AQ: 76,5 mg on day 2, [12–59] months, SP: 525 mg/AQ: 153 mg on day 0, SP: 0 mg/AQ: 153 mg on day 1, SP: 0 mg/AQ: 153 mg on day 2. Administration of the fisrt dose will be supervised by the community health workers while the second and third doses will be administered under supervision of the study field workers. Household members screening for malaria and treatment with the combination of dihydroartemisinin-piperaquine (DHA-PPQ), is a study-specific intervention which will be implemented in parallel with SMC over the four months SMC period. Household members with positive RDT test will be treated with single daily dose of DHAPPQ over three days in accordance with the national guideline for malaria cases management as follow: [5–8[Kg, DHA 20 mg/ PPQ 160 mg, one tablet/day, [8–11[Kg, DHA 20 mg/ PPQ 160 mg, 1 + 1/2 tablet /day, [11–17[Kg, DHA 40 mg/ PPQ 320 mg, one tablet/day, [17–25[Kg, DHA 40 mg/ PPQ 320 mg, 1 + 1/2 tablet /day and for [25–36[Kg, [36–60[Kg,, [60–80[Kg and ≥ 80 Kg, DHA 40 mg/ PPQ 320 mg, two, three, four and five tablets/day respectively. Enrolled children in both control and intervention arms will be followed for 24 months to fully cover two consecutive malaria transmission seasons, including two SMC cycles, and two dry seasons. However, the superiority will be measured over one season of intervention. The analysis will be repeated on year 2 to look at similar impact with potentially a difference in transmission. Then a pooled analysis of 2 years intervention will be conducted. Children will be actively followed-up with monthly visits during each of the two malaria transmission seasons (July–December). At each visit, physical examination will be performed and anthropometric parameters will be recorded, including weight, height, and Mid-Upper Arm Circumference (MUAC). Weight will be measured with SECA 874 scale (SECA company ltd., Germany). SHAKIR strips will be used for MUAC measurement. Blood smears will be collected by finger prick for hemoglobin (Hb) measurement, malaria Rapid Diagnostic Test (RDT), microscopy and additional drop of blood will also be spotted onto filter paper for later PCR analyses. In SMC+ screening and treatement of household members with standard RDT study arm, SD Bioline Malaria Ag P.f (HRP2/pLDH), (Standard Diagnostics, Inc., Korea) will be used. A HemoCue® 201+ will be used for photometric measurement of Hb in g/dL in the field. Thick and thin blood films will be stained with Giemsa 3% for 30 min and examined with light microscope Olympus CX23. Malaria slides will be double-read by two independent microscopists. In case of discrepencies a third reader will intervein and the arithmetic mean of the two nearest densities will be considered as final results. A smear will be declared negative if the examination of 100 thick-film fields does not reveal the presence of asexual parasites. The later PCR analyses will be the genotyping malaria-positive blood samples for amodiaquine resistance molecular markers in *pfcrt* and *pfmdr1*, sulfadoxine resistance marker in *pfdhps*, pyrimethamine resistance marker in *pfdhfr*, piperaquine resistance marker on Plasmepsin 2–3 and artemisinin resistance markers in the propeller domain of the Kelch 13 (K13) gene on malaria-positive samples. Samples will be genotyped using established protocols to detect molecular markers of antimalarial drug resistance. Briefly, DNA extracted from the dried blood spots will be used as the template for amplification by polymerase chain reaction (PCR). To detect nucleotide sequence polymorphisms, cleaned PCR products will be sent to a commercial service for Sanger sequencing. Single-nucleotide polymorphisms (SNPs) will be called by comparison with the reference sequences using validated bioinformatics workflows. Gene copy number amplifications will be detected using previously published protocols [20]. Children presenting any infection other than malaria will be diagnosed and treated appropriately according to national standard of care practices. After the malaria transmission season, the follow-up will be passive during the dry seasons (January–June). Parents / guardians of the enrolled children will be asked to contact the research team based at the peripheral health facilities of the study area in case their child is unwell. During these visits, children will be diagnosed and treated according to national standard of care practices. Nevertheless, all children will be actively seen on month 24 for a study end visit. Table 1 summarises the activites for the participant assessment and follow-up troughout the study (Table 1).

**Table 1:** Participant assessment and follow-up troughout the study in the health district of Nanoro, Burkina Faso

Time point	Year 1							Year 2							
	M1	M2	M3	M4	M5	M6	M7-M12 Passive Follow-up	M13	M14	M15	M16	M17	M18	M19–23 Passive Follow-up	M24 Close out visit
Written Consent	X														
Inclusion/Exclusion criteria	X														
SMC rounds	X	X	X	X				X	X	X	X				
Confirmation of SMC Drug intake	X	X	X	X				X	X	X	X				
Roommates screening and treatment	X	X	X	X				X	X	X	X				
Physical examination and Anthropometric indicators	X	X	X	X	X	X		X	X	X	X	X	X		X
Blood sample for Hb, RDT, Microscopy and filter spots	X	X	X	X	X	X		X	X	X	X	X	X	X	X
Adverse Events	X	X	X	X	X			X	X	X	X	X			

In terms of confidentiality, all medical records of the participants as well as information displaying their names or address details will be kept confidential at the IRSS/CRUN. However, these identifying information could be seen in case of need by the ethics committee for health research, or the regulatory authority in Burkina Faso to ensure that the study is properly run in accordiance with national regulations.

### Cost-effectiveness

A cost effectiveness module will be conducted in order to assess the incremental cost-effectiveness of screening and treatment of the household members of SMC-Children. To undertake cost effectiveness analysis, it is important that data on both costs and effectiveness are linked in such a way that only the incremental costs of those resources that produce the incremental effects are measured. The financial and economic costs (from the provider perspective) and cost-effectiveness of each intervention will be determined in order to better inform decision making. In addition, cost savings to the health systems due to malaria cases averted will be estimated. This cost effectiveness component will be carried out into two phases. In the first phase, the protocol and other study materials development will be guided by information collected via a literature review. In the second phase, the developed study materials will be used to collect the intended information by performing field interviews and observations.

Applying a micro-costing approach, information will be collected regarding the resources and costs associated with routine SMC and the new intervention. Screening and treatment of the household members of SMC-Children will be an “add-on” to existing SMC program. Costs for the introduction of the Screening and treatment of the household members of SMC-Children will be estimated for the two years of the intervention. The introduction costs refer to those investments that occur during the initial years of the introduction and typically include investments in additional training if needed, planning activities for a new intervention, sensitization etc. A cost calculation framework will be prepared in Excel, in which outputs will be generated (micro-costing) by multiplying p (unit cost of each item) with q (quantity of each item).

### Outcomes

The primary outcome is the incidence of clinical malaria evaluated within each year of the study in primary study participants i.e. children under SMC coverage. Secondary outcomes include (i) RDT and microscopy positivity rate from the beginning to the end of the follow-up, (ii) tolerance and safety i.e. the risk of adverse events (AEs) and serious adverse events (SAEs) after SMC drug uptake (AQSP) and after treatment with DHA-PPQ for household members (iii) prevalence of mutant alleles of *pfcrt*, *pfmdr1*, *pfdhfr*, and *pfdhps*, *plasmepsin*, and *K13* in malaria positive samples from the enrolment to the end of the follow-up periods, and (iv) adherence, (v) cost-effectiveness, and (vi) acceptability of the intervention which will be assessed qualitatively.

### Statistical analysis

The primary analysis will be a comparison of incidence rates between intervention and control treatment arms; incidence rate ratios and 95% confidence intervals will be also provided. Pooled incidence rate ratio and 95% CI will be calculated (if no interaction with time observed) using Poisson regression, adjusted for the year of study. The incidence of reported adverse events and serious adverse events will be summarized as a proportion of participants experiencing any of the events and a specific type of the event (defined by MEDRA) from the initiation of the treatment to day 30 after the last dose of the treatment. The AE incidence will be compared between study arms using Poisson regression. Prevalence of mutant alleles will be calculated as a proportion of samples with mutation for each study year and temporal trend will be explored using logistic regression and actual time of sample collection. Any comparisons between study arms including all participants will adjust for household clustering. A detailed statistical analysis plan will be produced before the database lock. Database will be locked at the final monitoring visit.

### Oversight and monitoring

Internal monitoring system by the NMCP is planned. This internal monitoring will be conducted to check for data collection accuracy and completeness of adverse events, and to verify the enrolment procedures by field workers. In addition, the project will be adequately monitored following a proposed monitoring plan. This will be led by an external experienced monitor hired from reference research center across the Greater Sahel research institutes. The monitor will verify the best conduct of the study through site visits and periodic phone calls with the principal investigator and other study staff. The monitor will have access to the study database that he/she will progressively review for consistency against source documents. At least four monitoring visits are planned.

Besides the monitoring and internal evaluation, an external intermediate scientific review will be performed. A scientific reviewing committee (SRC) will be set up before the study start. The members of the SRC will be selected from the Academy of Sciences of Burkina Faso, the Malaria task force of Burkina Faso and among international famous senior malariologists with strong experience in SMC intervention. The SRC will play the role of project monitoring group and will provide an external overview of all aspects of the study.

### Dissemination

Dissemination is critical for the adoption and translation of evidence-supported interventions into policy. Therefore, our consortium considers the dissemination component as an integral part of this study in order to ensure that our research findings will have the expected public health impact. This is particularly important in resource limiting settings where there are usually significant gaps or delays between the production of scientific evidences and their translation into policy. Taking this into account, the dissemination of the study results is planned not only through regular reports (implementation, progress, final), scientific papers and conference attendance, but also through meetings with various audiences of interest, including key stakeholders such as decision-makers and public health authorities; health workers; and members of the communities (including study participants and local community) directly impacted by the study results.

## Discussion

In Sahel countries, with high seasonal malaria transmission, SMC is an important strategy for reducing malaria burden. Despite the implementation of SMC intervention as a complement to multiple other malaria control measures, such as mass distribution of LLIN, malaria burden remains high in the most vulnerable groups including children younger than five years suggesting that the expected impact of this promising intervention is not reached. In order to achieve global malaria target by horizon 2030, development of innovative strategies to improve the efficacy of these existing malaria control measure is essential. This study aims to assess the impact of an improved SMC strategy adding roommates malaria screening and treatment with DHA-PPQ if positive. If confirmed to be beneficial, this strategy will contribute to breaking the cycle of continuous infection of children from parents and older siblings, as household members are not yet targeted by any specific intervention.

The choice for DHA-PPQ is driven by several reasons including its adoption as first line treatment for uncomplicated malaria in Burkina Faso [2, 21], its longer post-treatment prophylactic effect and most importantly its proven efficacy and safety in the area [22, 23].

Microscopy will be used in addition to RDT for the detection of malaria cases and asymptomatic parasitaemia in order mitigate the issue of false positive RDT due to the persistence of HRP2 antigen after a successful malaria treatment. Furthermore, microscopy will allow a better characterization of the detected cases in terms of parasitaemia, parasite species as well as gametocyte carriage which is critical for the transmission in the area.

SMC is delivered as a prevention measure during each malaria season but in this study the follow-up period is extended up to 24 months in order to cover two consecutive malaria seasons. The rationale for this extension include increasing the power to detect rare study outcomes such as severe malaria, related-mortality, rare safety signals with special interest to known severe reactions to SMC drugs and to DHAPPQ and to have an overview of the impact of the intervention overtime.

Superiority of the SMC + roommates malaria screening and treatment intervention over the routine implementation of SMC alone is expected. Therefore, the study will greatly contribute to identifying measures to reduce the malaria burden in Burkina Faso. By reducing morbidity and mortality, this study will have significant economic impact by promoting economic development and welfare in Burkina Faso, as malaria constitute an important threat to the development in the country.

We anticipate that holding SMC campaigns during the COVID-19 pandemic, combined with the fact that some parts of the country are prone to insecurity, may be challenging. However, it is important to stress that the 2020 SMC campaign has been fully implemented in the study area without any disturbance related to COVID-19 and/or security issue. In addition, the study area, Nanoro is located at 85 km from Ouagadougou, the capital city in the Central West Region of Burkina Faso, where we have not experienced any particular security issues so far.

Close monitoring of *P. falciparum* resistance markers to antimalarial drugs [24–27] will be conducted because the sustainability of any intervention, especially wide scale community-based intervention such as SMC, relies on the optimal efficacy of the drugs, especially as SMC induces a substantial drug pressure. Hence a close monitoring of the selection of resistance markers in the parasite population is paramount to describe the impact of the strategy.

Finally, this is a multidisciplinary collaborative research involving key stakeholders such as the WorldWide Antimalarial Resistance Network (WWARN), part of the Infectious Disease Data Observatory (IDDO) and policy makers such as the National Malaria Control Program (NMCP). The involvement of the NMCP will facilitate the rapid translation of the studyfindings into policy. In case of promising results, the NMCP will endorse the leadership of advocating with its financial and technical partners on the need to change the policy with the adoption and extension of this new strategy at national level. To better achieve this goal, a large-scale dissemination workshop will be organized by the NMCP to share the project findings. This workshop will bring together all key stakeholders of malaria policy decision making in Burkina Faso involving for example WHO, UNICEF, USAID, WAHO. The study could also be scaled up easily at regional level (through the regional Roll Back Malaria network) especially in other Sahelian countries with similar malaria epidemiological profile and where SMC is also implemented.

## Conclusion

This study will respond to a major public health concern by providing evidence of the efficacy of an innovative strategy to boost the impact of SMC intervention. This new approach should necessarily complement the existing ones to achieve the optimal impact in malaria control and elimination.

## Data Availability

Data supporting the conclusions of this article are included within the article and its additional files.

## Abbreviations

ACT: Artemisinin-based Combination Therapies
AE: Adverse Event
AQSP: Amodiaquine- Sulfadoxine-Pyrimethamine
CRUN: Clinicla Research Unit of Nanoro
DHAPPQ: Dihydroartemisinin-Piperaquine
DNA: Deoxyribonucleic acid
HDSS: Health and Demographic Surveillance System
HRP2: Histidin Rich Protein 2
IPTp: Intermittent Preventive Treatment in pregnancy
K13: Kelch 13
LLIN: Long-Lasting Insecticide treated Nets
NHD: Nanoro Health District
NMCP: National Malaria Control Program
PCR: polymerase chain reaction
Pf: *Plasmodium falciparum*
Pfcrt: *Plasmodium falciparum* chloroquine resistance transporter gene
Pfdhfr: *Plasmodium falciparum* dihydrofolate reductase
Pfdhps: *Plasmodium falciparum* Dihydropteroate synthetase
Pfmdr1: *Plasmodium falciparum* Multidrug resistance gene
RDT: Rapid Diagnostic Test
RST: Roommates Screening and Treatment
SAE: Serious Adverse Event
SMC: Seasonal malaria chemoprevention
SNP: Single Nucleotide Polymorphism
WWARN: WorldWide Antimalarial Resistance Network.
